# Parallel tiled Nussinov RNA folding loop nest generated using both dependence graph transitive closure and loop skewing

**DOI:** 10.1186/s12859-017-1707-8

**Published:** 2017-06-02

**Authors:** Marek Palkowski, Wlodzimierz Bielecki

**Affiliations:** West Pomeranian University of Technology, Faculty of Computer Science, Szczecin, 71-210 Poland

**Keywords:** RNA folding, Parallel biological computing, Loop tiling, Transitive closure, Loop skewing

## Abstract

**Background:**

RNA secondary structure prediction is a compute intensive task that lies at the core of several search algorithms in bioinformatics. Fortunately, the RNA folding approaches, such as the Nussinov base pair maximization, involve mathematical operations over affine control loops whose iteration space can be represented by the polyhedral model. Polyhedral compilation techniques have proven to be a powerful tool for optimization of dense array codes. However, classical affine loop nest transformations used with these techniques do not optimize effectively codes of dynamic programming of RNA structure predictions.

**Results:**

The purpose of this paper is to present a novel approach allowing for generation of a parallel tiled Nussinov RNA loop nest exposing significantly higher performance than that of known related code. This effect is achieved due to improving code locality and calculation parallelization. In order to improve code locality, we apply our previously published technique of automatic loop nest tiling to all the three loops of the Nussinov loop nest. This approach first forms original rectangular 3D tiles and then corrects them to establish their validity by means of applying the transitive closure of a dependence graph. To produce parallel code, we apply the loop skewing technique to a tiled Nussinov loop nest.

**Conclusions:**

The technique is implemented as a part of the publicly available polyhedral source-to-source TRACO compiler. Generated code was run on modern Intel multi-core processors and coprocessors. We present the speed-up factor of generated Nussinov RNA parallel code and demonstrate that it is considerably faster than related codes in which only the two outer loops of the Nussinov loop nest are tiled.

## Background

RNA secondary structure prediction is an important ongoing problem in bioinformatics. RNA provides a mechanism to copy the genetic information of DNA and can catalyze various biological reactions. RNA folding is the process by which a linear ribonucleic acid molecule acquires secondary structure through intra-molecular interactions.

Algorithms to make predictions of the structure of single RNA molecules use empirical models to estimate the free energies of folded structures. This paper focuses on the base pair maximization algorithm developed by Nussinov [1], which predicts RNA secondary structure in a computationally efficient way. Given an RNA sequence *x*_1_,*x*_2_,…,*x*_*n*_, where *x*_*i*_ is a nucleotide from the alphabet {G (guanine), A (adenine), U (uracil), C (cytosine)}, Nussinov’s algorithm solves the problem of RNA non-crossing secondary structure prediction by means of computing the maximum number of base pairs for subsequences *x*_*i*_,…,*x*_*j*_, starting with subsequences of length 1 and building upwards, storing the result of each subsequence in a dynamic programming array.

The following Nussinov recursion *S*(*i,j*) is defined over the region 1≤ *i*<*j*≤*N* as

1 $$ \begin{array}{ll} S(i,j) &= max(S(i+1, j-1) + \delta(i,j), \\ &\underset {i \leq k < j} {max} (S(i, k) + S(k + 1, j))),  \end{array}  $$

and zero elsewhere, where *S* is the *N*×*N* Nussinov matrix, and *δ*(*i,j*) is the function which returns 1 if (*x*_*i*_,*x*_*j*_) is an AU, GC or GU pair and *i*<*j*, or 0 otherwise.

Nussinov’s algorithm is within nonserial polyadic dynamic programming (NPDP). The term nonserial polyadic stands for another family of dynamic programming (DP) with nonuniform data dependences, which is more difficult to be optimized [2].

On modern computer architectures, the cost of moving data from main memory is orders of magnitude higher than the cost of computation. Improving data locality and extracting loop nest parallelism of NPDP are still challenging tasks, although a number of authors have developed theoretical approaches to accelerating NPDP codes for RNA folding [3–8].

Fortunately, the Nussinov recursion involves mathematical operations over affine control loops whose iteration space can be represented by the polyhedral model [9]. In this paper, we consider a formulation that is suitable for automatically producing parallel and tiled program loop nests from the dependence structure of the program (as would be used in an automatic optimizing compiler).

Loop tiling, or blocking, is a key transformation used for both coarsening the granularity of parallelism and improving code locality. Smaller blocks of loop nest statement instances in a loop nest iteration space (tiles) can improve cache line utilization and avoid false sharing. On the basis of a valid schedule of tiles, parallel coarse-grained code can be generated.

To our best knowledge, well-known loop nest tiling techniques are based on linear or affine transformations [10–13]. However, only the two outer loops from the three ones of the Nussinov code can be tiled by means of standard tiling algorithms implemented in polyhedral tools [14]. For example, the state-of-the-art compiler, Pluto [10], extracting and applying affine transformations, is able to tile and parallelize the two outer loops of the considered Nussinov code and is not able to tile the innermost loop. The iterations of this loop can be executed only in serial order that prevents enhancing code locality and parallelism degree.

Moreover, classical affine transformations have commonly known limitations [9, 14, 15], which complicate extraction of available parallelism and locality improvement in NPDP codes. Mullapudi and Bondhugula presented dynamic tiling for Zuker’s optimal RNA folding^1^ in paper [9]. They have explored techniques for tiling codes that lie outside the domain of standard tiling techniques. 3D iterative tiling for dynamic scheduling is calculated by means of reduction chains. Operations along each chain find maximum and can be reordered to eliminate cycles. Their approach involves dynamic scheduling of tiles, rather than the generation of a static schedule. At this time, a precise characterization of the relative domains of this technique is not available.

Wonnacott et al. introduced 3D tiling of “mostly-tileable” loop nests of the Nusinov algorithm in the paper [14]. The “mostly-tileable” term means the iteration space is dominated by non-problematic iterations (iterations of loops ’*i*’ and ’*j*’). This approach tiles non-problematic iterations with classic tiling strategies while problematic iterations of loop (’*k*’) are peeled off and executed later. Generated code is serial and the authors do not present any parallelization of this code.

Rizk et al. [16] provide an approach to produce efficient GPU code for RNA folding, but they do not consider any loop nest tiling. Tang et al. [17] presented the Pochoir compiler for automatic parallelization and cache performance optimization of stencil computations. Pochoir computes the optimal cost of aligning a pair of DNA or RNA sequences by means of Gotoh’s algorithm. It transforms computation to obtain diamond-shaped grid that can be evaluated as a stencil, but it can tile only two of the three loops of original code. Stivala et al. [18] describe a lock-free algorithm for parallel dynamic programming. However, code locality improvement is not considered.

Paper [15] introduces a new technique to generate parallel code applying the power *k* of a relation representing a dependence graph, but that paper does not consider generation of tiled code and does not concern any RNA folding. Paper [19] considers runtime scheduling of RNA folding for untiled program loops with known bounds.

Motivated by the deficiency of the mentioned techniques, we developed and present in this paper a novel approach for tiling and parallelization of the Nussinov loop nest. To generate valid tiles in all three dimensions, we apply the exact transitive closure of loop nest dependence graphs. It allows for generating target tiles such that there is no cycle in a corresponding inter-tile dependence graph. It is well-known that for such a case, a valid schedule of target tiles exists, i.e., a valid serial or parallel tiled code can be generated [9]. Such a tiling can be applied to bands of original loops not being fully permutable. To parallelize generated serial tiled code, we use the loop skewing transformation and prove its application validity.

## Methods

### Brief introduction

An introduced approach uses the dependence analysis proposed by Pugh and Wonnacott [20] where dependences are represented by relations with constraints defined by means of the Presburger arithmetic using logical and existential operators. A dependence relation is a tuple relation of the form [*input list*] →[*output list*]: *formula*, where *input list* and *output list* are the lists of variables and/or expressions used to describe input and output tuples and *formula* describes the constraints imposed upon *input list* and *output list*. Such a relation is a mathematical representation of a data dependence graph whose vertices correspond to loop statement instances while edges connect dependent instances. The input and output tuples of a relation represent dependence sources and destinations, respectively; the relation constraints specify instances which are dependent.

Standard operations on relations and sets are used, such as intersection (∩), union (∪), difference (−), domain (dom *R*), range (ran *R*), relation application (*S* ^′^ = *R*(*S*): *e* ^′^∈*S* ^′^ iff exists *e* s.t. *e* →*e* ^′^∈*R*, *e* ∈*S*). In detail, the description of these operations is presented in papers [20, 21].

The positive transitive closure for a given lexicographically forward relation *R*, *R* ^+^, is defined as follows [21]:

$$\begin{aligned} R^{+}&=\{e\rightarrow e':\ e\rightarrow e'\in R \ \vee \\ &\quad\exists e^{\prime\prime} s.t.\ e\rightarrow e^{\prime\prime} \in R \ \land \ e^{\prime\prime}\rightarrow e'\in R^{+}\}. \end{aligned} $$

It describes which vertices *e* ^′^ in a dependence graph (represented by relation *R*) are connected directly or transitively with vertex *e*.

**Fig. 1:**
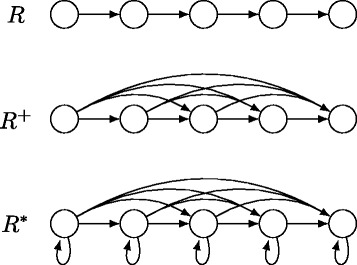
Transitive closure. An example of dependence relation *R*, positive transitive closure *R* ^+^, and transitive closure *R* ^∗^

Transitive closure, R*, is defined as below:

$$R^{*} = R^{+} \cup I, $$

where *I* is the identity relation. It describes the same connections in a dependence graph (represented by *R*) that *R* ^+^ does plus connections of each vertex with itself. Figure 1 presents *R* ^+^ and *R* ^∗^ in a graphical way.

In the sequential loop nest, the iteration *i* executes before *j* if *i* is *lexicographically less* than *j*, denoted as

2 $$  i \prec j, i.e., i_{1}<j_{1} \lor \exists k \geq 1 : i_{k} <j_{k} \land i_{t}=j_{t},\ for \ t<k.  $$

A *schedule* is a function $\sigma : LD \rightarrow \mathbb {Z}$ which assigns a discrete time of execution to each loop nest statement instance or tile. A schedule is *valid* if for each pair of dependent statement instances, *s*_1_(*I*) and *s*_2_(*J*), satisfying the condition *s*_1_(*I*)≺*s*_2_(*J*), the condition *σ*(*s*_1_(*I*))<*σ*(*s*_2_(*J*)) holds true, i.e. the dependences are preserved when statement instances are executed in an increasing order of schedule times.



### The Nussinov loop nest

**Fig. 2:**
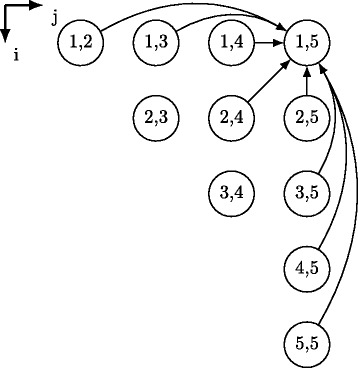
Cell dependences. Nussinov’s loop nest dependences for one iteration (*i*=1,*j*=5); iteration (*i*=1,*j*=5) depends on three adjacent iterations and five non-local ones

The Nussinov recurrence is challenging to accelerate because of its non-local dependency structure shown in Fig. 2. Cell *S*(*i,j*) is depended to adjacent cells of the dynamic programming matrix as well as to non-local cells. These non-local dependences are affine, that is, *S*(*i,j*) depends on other cells *S*(*r,s*) such that the differences *i*–*r* or *j*–*s* are not constant but rather depend on *i* and *j*. Therefore, the Nussinov data dependences result in a nonuniform structure [5]. Equation 1 leads directly to the form of the $\mathcal {O}(n^{3})$ Nussinov loop nest presented in Listing 1. The loop nest is imperfectly-nested and is comprised of two statements, *s0* and *s1*.

The following sub-section discusses how to generate serial tile code by means of the transitive closure of dependence graphs.

### Loop nest tiling based on the transitive closure of dependence graphs

To generate valid tiled code, we apply the approach presented in paper [22] based on the transitive closure of dependence graphs. We briefly present the steps of that technique for tiling the Nussinov loop nest. Dependence relations for this loop nest, including non-uniform ones, can be extracted with Petit (the Omega project dependence analyser) [20] and they are presented below.

$$ R = \left\{ \begin{array}{l} s0\rightarrow s0 : \{[i,j,k] \rightarrow [i,j',j-i] : j < j' < N \wedge \\ \qquad 0 \leq k \wedge i+k < j \wedge \ 0 \leq i\} \ \cup \\ \qquad \{[i,j,k] \rightarrow [i',j,i-i'-1] : \\ \qquad 0 \ \leq i'< i \wedge j < N \wedge \ 0 \leq k \wedge i+k < j\} \ \cup \\ \qquad \{[i,j,k] \rightarrow[i,j,k'] : 0 \leq k < k' \wedge \ j < N \\ \qquad \wedge 0 \leq i \wedge i+k' < j\} \\ s0\rightarrow s1 : \{[i,j,k]\rightarrow [i-1,j+1] : j \leq N-2 \ \wedge \\ \qquad \ 0 \leq k \wedge i+k < j \ \wedge 1 \leq i\} \ \cup \\ \qquad \{[i,j,k] \rightarrow [i,j] : j < N \wedge 0 \leq k \ \wedge \\ \qquad i+k < j \wedge 0 \leq i\}\\ s1\rightarrow s0 : \{[i,j] \rightarrow [i,j',j-i] : 0 \leq i < j < j' < N\}\\ \qquad \cup \{[i,j] \rightarrow [i',j,i-i'-1] : \\ \qquad 0 \leq i' < i < j < N\}\\ s1\rightarrow s1 : \{[i,j] \rightarrow [i-1,j+1] : 1 \leq i < j \leq N-2 \}. \end{array}\right. $$

Next, we calculate the exact transitive closure of the union of all dependence relations, *R* ^+^, applying the modified Floyd-Warshall algorithm [23]. For brevity, we skip the mathematical representation of *R* ^+^.

Let vector ***I***=(*i,j,k*)^*T*^ represent indices of the Nussinov loop nest, vector ***B***=(*b*_1_,*b*_2_,*b*_3_)^*T*^ define an original tile size, vectors ***II***=(*ii,jj,kk*)^*T*^ and ***II*** ^***′***^=(*iip,jjp,kkp*)^*T*^ specify tile identifiers. Each tile identifier is represented with a non-negative integer, i.e., the constraints ***II***≥ 0 and ***II*** ^***′***^≥ 0 have to be satisfied.

Below, the mathematical representation of original rectangular tiles for the Nussinov loop nest with the tile size defined with vector ***B*** is presented.

$$TILE = \left\{ \begin{array}{l} i : N-1-b_{1}* ii \geq i \geq max(-b_{1}*(ii+1),\\ \quad N-1) \wedge ii \geq 0 \\ j : b_{2} * jj +i+1 \leq j \leq min(b_{2}*(jj+1) + 1,\\ \quad N-1) \wedge jj \geq 0\\ k : \left\{\begin{array}{l} s0 : b_{3} * kk \leq k \leq min(b_{3}*(kk+1)-1, \\ \quad j-i-1) \wedge kk \geq 0\\ s1 : k = 0. \end{array}\right. \end{array}\right. $$

Let us note that for index *i*, the constraints are defined inversely because the value of index *i* is decremented.

For the tile identifiers, we define constraints, *CONSTR*(***II***,***B***), which have to be satisfied for given values *b*1, *b*2, *b*3, defining a tile size, and parameter *N* specifying the upper loop index bound.

3 $$ CONSTR(\boldsymbol{II}, \boldsymbol{B}) = \left\{\begin{array}{l} ii, b_{1} : N-1 - b1*ii >= 0\\ jj, b_{2} : (i+1) + b2 * jj <= N-1\\ kk, b_{3} : b3*kk + 0 <= j-i-1. \end{array}\right.  $$

In accordance with formula (2), we present below the lexicographical ordering ***II***≺***II*** ^***′***^ on vectors ***II***, ***II*** ^***′***^ defining tile identifiers as follows.

$$ \boldsymbol{II}^{\boldsymbol{\prime}} \prec \boldsymbol{II} = \left\{\begin{array}{l} s0 : \left\{\begin{array}{l} s0: ii > iip \vee (ii = iip \wedge jj > jjp) \ \vee \\ \quad (ii=iip \wedge jj=jjp \wedge kk > kkp))\\ s1: ii > iip \vee (ii = iip \wedge jj > jjp) \end{array}\right.\\ s1: \left\{\begin{array}{l} s0: ii > iip \vee (ii = iip \wedge jj > jjp) \ \vee\\ \quad\ (ii=iip \wedge jj=jjp))\\ s1: ii > iip \vee (ii = iip \wedge jj > jjp). \end{array}\right. \end{array}\right. $$

Next, we build sets *TILE_LT* and *TILE_GT* that are the unions of all the tiles whose identifiers are lexicographically less and greater than that of *TILE*(***II***, ***B***), respectively:

*TILE*_*LT*(*GT*)={[***I***]| ∃ ***II*** ^***′***^:***II*** ^***′***^≺(≻)***II***∧***II***≥0∧

*CONSTR*(***II***,***B***) ∧***II*** ^***′***^≥0 ∧*CONSTR*(***II*** ^***′***^,***B***) ∧ ***I***∈*TILE*(***II*** ^***′***^,***B***)}.

Using the exact form of *R* ^+^, we calculate set, *TILE*_*ITR*, as follows.

$$TILE{\_}ITR = TILE - R^{+}(TILE{\_}GT). $$

This set does not include any invalid dependence target, i.e., it does not include any dependence target whose source is within set *TILE_GT.*

The following set

$$\begin{aligned} TVLD{\_}LT &= (R^{+}(TILE{\_}ITR) \cap TILE{\_}LT) \\ &\quad- R^{+}(TILE{\_}GT) \end{aligned} $$

includes all the iterations that i) belong to the tiles whose identifiers are lexicographically less than that of set *TILE_ITR*, ii) are the targets of the dependences whose sources are contained in set *TILE_ITR*, and iii) are not any target of a dependence whose source belong to set *TILE_GT*.

Target valid tiles are defined by the following set

$$ TILE{\_}VLD = TILE{\_}ITR \cup TVLD{\_}LT. $$

To generate serial tiled code, we first form set *TILE_VLD_EXT* by means of inserting i) into the first positions of the tuple of set *TILE_VLD* elements of vector ***II*** : *ii,jj,kk*; ii) into the constraints of set *TILE_VLD* the constraints defining tile identifiers ***II***≥ 0 and *CONSTR*(***II***,***B***).

The following step is to use the original schedule of the original Nussinov loop nest statement instances, *SCHED*_*ORIG*, to form a target set allowing for re-generation of serial valid code. The original schedule can be extracted by means of the Clan tool [24] and is as shown below.

$$SCHED{\_}ORIG = \left\{\begin{array}{l} s0 : 0,i,0,j,0,k\\ s1 : 0,i,0,j,1,k.\end{array}\right. $$

Next we enlarge that schedule with indices *ii,jj,kk* (responsible for tile identifiers) repeating the same sequence of elements as that for indices *i,j,k* in the original schedule to get the following schedule.

$$SCHED=\left\{\begin{array}{l} s0 : 0,ii,0,jj,0,kk,0,i,0,j,0,k\\ s1 : \left\{\begin{array}{l} s0 : 0,ii,0,jj,1,kk,0,i,0,j,0,k\\ s1 : 0,ii,0,jj,1,kk,0,i,0,j,1,k. \end{array}\right. \end{array}\right. $$

Let us note that tiles, formed for statement *s0*, include only instances of statement *s0*, while those generated for statement *s1* comprise instances of both statement *s0* and statement *s1*.

In the next step, we form relation, *Rmap*_*s*0_, for the sub-set of set *TILE*_*VLD*_*EXT* representing tiles for statement *s*0, as follows

$$Rmap_{s0}= \left\{\begin{array}{l} \ \ TILE{\_}s0 \ [ii,jj,kk] \rightarrow \\ \qquad [0, ii, 0, jj, 0, kk, 0, i, 0, j, 0, k]\end{array}\right\}, $$

and relation, *Rmap*_*s*1_, for the sub-set of set *TILE*_*VLD*_*EXT* representing tiles for statement *s*1, as follows

$$Rmap_{s1}= \left\{\begin{array}{l} \ \ \ \ TILE{\_}s0 \ [ii,jj,kk] \ \rightarrow \\ \qquad [0, ii, 0, jj, 1, kk, 0, i, 0, j, 0, k]; \\ \ \ TILE{\_}s1 \ [ii,jj,kk] \rightarrow \\ \qquad [0, ii, 0, jj, 1, kk, 0, i, 0, j, 1, k]\end{array}\right\}, $$

and finally, form target set, *TILE*_*VLD*_*EXT* ^′^, as bellow

$$TILE{\_}VLD{\_}EXT' = Rmap(TILE{\_}VLD{\_}EXT), $$

where *Rmap*=*Rmap*_*s*0_∪*Rmap*_*s*1_.

Sequential tiled code is generated by means of applying the isl AST code generator [25] allowing for scanning elements of set *TILE*_*VLD*_*EXT* ^′^ in lexicographic order.

### Tiled code parallelization

To parallelize generated serial tiled code, we apply the well-known loop skewing transformation [26]. Loop skewing is a transformation that has been used to remap an iteration space by creating a new loop whose index is a linear combination of two or more loop indices. This results in code whose outermost loop is serial while the other loops can be parallelized.

**Fig. 3:**
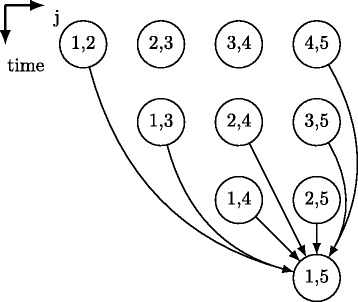
Loop skewing. Scheduling for Nussinov’s recurrence cells. Cells lying on each horizontal line are independent and can be run in parallel; the vertical coordinate represents time partitions to be enumerated serially

We use the following skewing transformation: *ii* ^′^=*ii*+*jj*, where *ii* ^′^ is the new loop index, *ii,jj* are the indices of the first two loops in tiled code. Figure 3 illustrates the loop skewing technique applying to the Nussinov loop nest. Iterations lying on each horizontal line can be executed in parallel while time partitions should be enumerated serially.

To apply the loop skewing transformation, we create the following relation

$$\begin{aligned} R{\_}{SCHED} &= \{[0,ii',0,jj,\ldots,0,i,0,j,\ldots] \rightarrow \\ &\left[0, ii + jj, 0, jj, \ldots, 0, -i, 0, j,\ldots\right]: \\ & constraints\ of\ set \ TILE{\_}VLD{\_}EXT'\ \}, \end{aligned} $$

and apply it to set *TILE*_*VLD*_*EXT* ^′^.

Applying the loop skewing transformation is not always valid. To prove the validity of this transformation applied to generated serial tiled code, we form the following relation, *R*_*VALID*, which checks whether all original inter-tile dependences will be respected in parallel code.

$$\begin{array}{l} R{\_}{VALID} = \{[\boldsymbol{II}] \rightarrow [\boldsymbol{JJ}]|\ \exists\ \boldsymbol{I}, \boldsymbol{J}: \\ \underbrace{\boldsymbol{I} \in domain \ R\ \wedge \ \boldsymbol{J} = R(\boldsymbol{I})\ }_{\text{(*)}}\ \wedge\ \\ \underbrace{\boldsymbol{I} \in\ TILE(\boldsymbol{II}) \wedge\ \boldsymbol{J} \in\ TILE(\boldsymbol{JJ})}_{\text{(**)}}\ \wedge \\ \underbrace{R{\_}SCHED(\boldsymbol{II})\ \succeq \ R{\_}SCHED(\boldsymbol{JJ})}_{\text{(***)}} \}, \end{array} $$

where:

(*) means that ***J*** is the destination of the dependence whose source is ***I***,

(**) means that ***I***,***J*** belong to the tiles with identifiers ***II*** and ***JJ***, respectively,

(***) means that the schedule time of tile ***II*** is greater or the same as that of tile ***JJ***, i.e., the schedule is invalid because the dependence ***I***→***J*** is not respected.

**Fig. 4:**
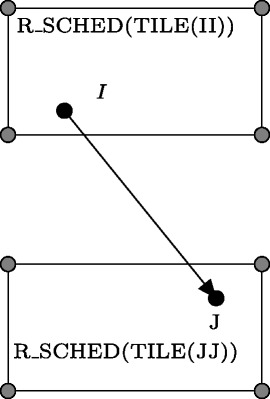
Illustration of an invalid schedule. Vectors *I* and *J* represent the source and destination of a dependence, respectively. TILE(*II*) is scheduled to run after (lexicographically greater) TILE(*JJ)*



This relation returns the empty set when all original inter-tile dependences are respected, otherwise it represents all the pairs of the tile identifiers for which original ones are not respected. Figure 4 presents the case of an invalid schedule, where ***I*** and ***J*** are vectors representing the source and destination of a dependence, respectively, within the tiles with identifiers ***II*** and ***JJ***. Relation *R*_*VALID* is empty for the generated serial tiled Nussinov code, this proves the validity of applying the loop skewing transformation.

Target pseudo-code is generated by means of applying the isl AST code generator [25] allowing for scanning elements of set *R*_*SCHED*(*TILE*_*VLD*_*EXT* ^′^) in lexicographic order. Then we postprocess this code replacing pseudo-statements for the original loop nest statements and insert the work-sharing OpenMP *parallel for* pragmas [27] before the second loop in the generated code to make it parallel. Listing 2 presents the target code for the Nussinov loop nest (Listing 1) tiled with the tiles of the size 16x16x16. The first loop in this code enumerates serially time partitions while the second one scans all the tiles to be executed in parallel for a given time defined with the first loop.

## Results and discussion

The presented approach has been implemented as a part of the polyhedral TRACO compiler^2^. It takes on input an original loop nest in the C language, a tile size, and affine transformations for each loop nest statement to parallelize serial tiled code. Then TRACO generates serial valid tiled code and checks whether the affine transformations are valid by means of calculating relation *R*_*VALID*. If so, parallel tiled code is generated.

All parallel Nussinov tiled codes were generated by means of the Intel C++ Compiler (*icc* 17.0.1) with the -O3 flag of optimization.

This section presents speed-up of generated parallel tiled code. To carry out experiments, we used machines with two processors Intel Xeon E5-2699 v3 (3.6 Ghz, 32 cores, 45MB Cache), four coprocessors Intel Xeon Phi 7120P (1.238 GHz, 61 cores, 30.5 MB Cache), and 128 GB RAM.

Problem sizes 2200 and 5000 were chosen because they are the average and the longest lengths of randomly generated RNA strands (from the {ACGU} alphabet) in human body to illustrate any additional advantages for medium and larger instances, respectively [14]. Furthermore, we used several mRNAs and lncRNAs from the NCBI database^3^ for homo sapiens. Analyzing the program code, we expected there should be no difference, performance wise, between actual sequences versus randomly generated sequences. To confirm this fact, we measured the summary time of calling bonding function *δ*(*i,j*). It takes less than 0.2 percent of the whole tiled code running time regardless of the sequence type, for example, 0.017 seconds for the problem size equal to 5000 (over 12 mln calls) on an Intel Xeon E5-2699 v3 platform. It can be therefore concluded that the studied algorithm performance does not change based on the strings themselves, but it depends on the size of a string.

For generated tiled code, we empirically recognized that the best tile size is 16x16x16 and the most efficient work-sharing is achieved by applying the OpenMP *for* directive [27] with the dynamic scheduling of loop iterations and the chunk size equal to 1.

**Fig. 5:**
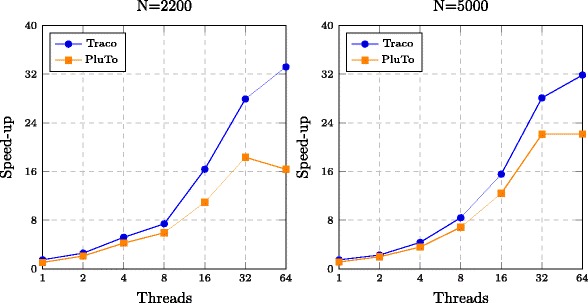
Speed-up of parallel codes using two 32-core processors Intel Xeon E5-2699 v3. The horizontal coordinate represents number of threads and the vertical one shows the speedup of codes generated with the TRACO and PluTo compilers for two problem sizes of RNA folding

**Fig. 6:**
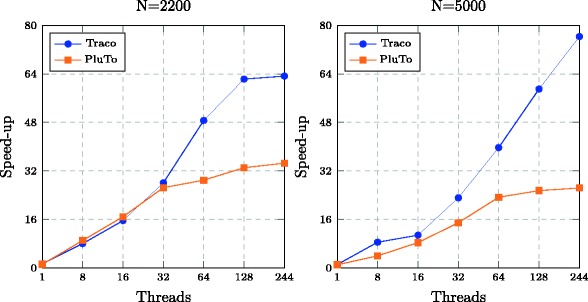
Speed-up of parallel codes using four 61-core coprocessors Intel Xeon Phi 7120P. The horizontal coordinate represents number of threads and the vertical one shows the speedup of codes generated with the TRACO and PluTo compilers for two problem sizes of RNA folding

Table 1 presents the execution times of the serial original and parallel tiled Nussinov loop nest from one to 64 threads for Intel Xeon E5-2699 v3 processors and from one to 244 threads for Intel Xeon Phi 7120P coprocessors. As we can see, for all cases, the execution time of the tiled codes is shorter than that of the original code and it reduces with increasing the number of threads. Speed-up is illustrated in Figs. 5 and 6 in a graphical way for multi-core processors and coprocessors, respectively.

**Table 1:** Execution times (in seconds) of the tiled Nussinov loop nest

Platform	Threads	Times	
		N=2200	N=5000
Intel Xeon	1 (original)	12.28	334.32
E5-2699 v3	1	8.25	225.23
	2	4.76	147.30
	4	2.37	76.79
	8	1.66	39.81
	16	0.75	21.49
	32	0.44	11.90
	64	0.37	10.50
Intel Xeon	1 (original)	235.38	2879.66
Phi 7120P	1	166.92	2556.65
	8	29.29	339.15
	16	15.09	266.34
	32	8.38	124.51
	64	4.84	72.56
	128	3.78	48.81
	244	3.72	37.75

Those figures also present the speed-up of parallel 2D tiled code produced with the state-of-the-art Pluto+ [28] optimizing compiler, which does not enable to tile the third loop in the Nussinov loop nest^4^. From Figs. 5 and 6, we may conclude that the tiled code generated with the proposed approach outperforms that generated with standard affine transformations extracted and applied with Pluto+ for both Intel multi-core processors and coprocessors.

The parallel code presented in the paper is not synchronization free (to our best knowledge, there does not exist any synchronization-free code for Nussinov’s loop nest), after each parallel iteration multiple tasks must be synchronized. Synchronization usually involves waiting by at least one task, and can therefore cause a parallel applications wall clock execution time to increase, i.e., it introduces parallel program overhead. Any time one task spends waiting for another is considered synchronization overhead. Synchronization overhead grows with increasing the number of synchronization events and the number of threads and tends to grow rapidly (in a non-linear manner) as the number of tasks in a parallel job increases, it is the most important factor in obtaining good scaling behavior for the parallel program. Synchronization overhead leads to non-linear character of speed-up when the numbers of threads grows (see Figs. 5 and 6). When the number of threads are less than 16, the code presented in the paper and that generated with PLUTO, have comparable synchronization overhead and locality, but for the number of threads more than 16, our code has less synchronization overhead and better locality that results in higher speed-up.

It is worth noting that the generated tiled serial code has improved locality in comparison with that of the serial original code. This results in about 1.5 and 1.4 higher serial tiled code performance for the used Intel muti-core processors and co-processors, respectively. Below, we compare the speed-up achieved for the tiled code generated by the presented technique with that of related code.

In paper [7], the authors write: “We have developed GTfold, a parallel and multicore code for predicting RNA secondary structures that achieves 19.8 fold speedups over the current best sequential program”. This speed-up is achieved on 32 threads. The code, presented in our paper, outperforms this code (for 32 threads, it yields 28.1 speed-up for the problem size equal to 5000). We also present speed-up for 64 threads for an Intel Xeon E5-2699 v3 platform and from one to 244 threads for Intel Xeon Phi 7120P coprocessors. The higher performance of our code is achieved due to applying loop nest tiling.

Rizk et al. [16] provide an efficient GPU code for RNA folding, but they do not consider any loop nest tiling. The authors give a table which shows that the maximal speedup, using a graphical card GTX280, is 33.1. Applying Intel Xeon Phi 7120P coprocessors for running our code, we reach the maximal speed-up 75.6 for 244 threads (the problem size is equal to 5000). This demonstrates that tiling allows for considerable improving code locality that leads to significant increasing parallel code speed-up.

Pochoir [17] computes the optimal cost of aligning a pair of DNA or RNA sequences by means of diamond-shaped grid that can be evaluated as a stencil, but it can tile only two of the three loops of original code, i.e., tiled code is of maximum 2-d dimension. This results in only 4.5 speedup of the RNA code generated with Pochoir on 12 cores – the maximal number of cores that the authors examined.

Summing up, we conclude that the presented approach allows for generation of a parallel tiled Nussinov loop nest which considerable reduces execution time in comparison with related codes. The code presented in our paper is dedicated to be run on high performance computer systems with the large number of cores. Since the number of cores tends to grow, in our opinion, the presented code is very actual because it has improved scalability and can be run on computer systems with the large number of cores.

## Conclusion

The paper presents automatic tiling and parallelization of the Nussinov program loop nest. The transitive closure of dependence graphs is used to tile this code, whereas for extracting parallelism in the tiled loop nest, the loop skewing transformation is applied, which is within the affine transformation framework. To the best of our knowledge, the presented approach is the first attempt to generate static parallel 3D tiled code for Nussinov’s prediction. An experimental study demonstrates significant parallel tiled code speed-up achieved on modern multi-core computer systems.

The presented approach is an important starting point for future research aimed at effective tiling and parallelization of other NPDP codes, in particular the detailed energy models used by Zuker’s algorithm.

We are going to examine how the presented approach based on both the transitive closure of dependence graph and affine transformations can be applied to tile and parallelize other important applications of bioinformatics.

## Endnotes

^1^ Zuker’s algorithm has the same dependence patterns as Nussinov’s algorithm [9].

^2^ http://traco.sourceforge.net

^3^ https://www.ncbi.nlm.nih.gov/

^4^ Pluto 0.11.4 BETA and Pluto+ generate the same tiled code for the Nussinov loop nest.

## Abbreviations

AST: Abstract syntax tree
DP: Dynamic programming
GPU: Graphics processing unit
NPDP: Nonserial polyadic dynamic programming
